# Silent Migration of an Intrauterine Device into the Peritoneum: A Case Report

**DOI:** 10.7759/cureus.95176

**Published:** 2025-10-22

**Authors:** Morris A Simwa, Satbir Karwal, Sumayyah Ibrahim, Anisha Dave, Ahmed Alsobhi, Edna Mensah, Mahmoud Hani

**Affiliations:** 1 General Medicine, University Hospital Southampton NHS Foundation Trust, Southampton, GBR; 2 Obstetrics and Gynecology, Shree Swaminarayan Hospital, Nairobi, KEN; 3 General Medicine, Aberdeen Royal Infirmary, Aberdeen, GBR; 4 General Practice, Shree Swaminarayan Hospital, Nairobi, KEN; 5 Cardiology, University Hospital Southampton NHS Foundation Trust, Southampton, GBR; 6 Internal Medicine, University Hospital Southampton NHS Foundation Trust, Southampton, GBR

**Keywords:** device migration, displaced iud, explorative laparotomy, gynae, intrauterine device migration, iud, migrated iud, omental involvement, uterine perforation, uterine perforation by intrauterine device

## Abstract

Uterine perforation is an uncommon but potentially adverse outcome of intrauterine device (IUD) placement. It often occurs around the time of insertion and may go unnoticed, though delayed perforation can also present months or years later. Diagnosis is typically suspected when IUD strings are not visualized on vaginal examination and confirmed by imaging demonstrating displacement, perforation, or migration. Definitive management requires device removal. We report the case of a 34-year-old woman with silent migration of a levonorgestrel-releasing IUD into the peritoneal cavity. IUD displacement was incidentally noted during a routine Papanicolaou (Pap) test six months after insertion when the strings were not visualized. A pelvic ultrasound done at that time reported an IUD embedded in the uterine wall without serosal perforation. Unfortunately, delayed follow-up, partly due to financial limitations, caused further migration. A repeat ultrasound and pelvic X-ray performed 11 months after insertion confirmed intraperitoneal displacement. She remained asymptomatic throughout this period. The IUD was ultimately retrieved by laparotomy, where it was found in the peritoneum, loosely adherent to the greater omentum. This case underscores the potential for asymptomatic migration of a levonorgestrel-releasing IUD, the risks associated with delayed intervention, and the influence of financial and resource limitations on timely diagnosis and optimal management.

## Introduction

Intrauterine devices (IUDs) are widely used contraceptives due to their high effectiveness, low cost, and extended duration of action [1]. Globally, approximately 14.3% of women of reproductive age use IUDs, though the prevalence varies markedly by region [2]. Complications may occur with insertion, including pain, infection, abnormal bleeding, expulsion, and uterine perforation [3]. Perforation is rare, with an incidence of 0.1%, and often goes unrecognized at the time of placement [4]. Symptoms depend on the final site of the IUD migration, which may vary from the pouch of Douglas to the mesentery, colon, or urinary bladder. Some patients present with non-specific symptoms or may be asymptomatic, delaying diagnosis and management [5]. In such patients, displacement is often first suspected when strings are absent on vaginal examination or the IUD is incidentally noted on imaging performed for other reasons. Confirmation of device migration can be achieved with ultrasound, abdominal X-ray, or computed tomography, with the latter offering the most accurate localization [5,6]. Minimally invasive techniques such as laparoscopy are the gold standard for extrauterine IUD retrieval, offering lower morbidity and shorter hospital stays, unless extensive adhesions or other complicating factors necessitate laparotomy [7]. The high upfront costs and dependence on specialized equipment often make minimally invasive approaches less feasible in resource-limited settings [8]. Our case highlights the silent migration of a levonorgestrel-releasing IUD, the risks of delayed intervention, and the impact of financial and resource limitations on timely diagnosis and optimal management.

## Case presentation

A 34-year-old woman with two previous pregnancies, including one cesarean delivery seven years earlier, underwent successful insertion of a levonorgestrel-releasing 52 mg IUD (Mirena) at a general clinic. She subsequently experienced two days of colicky suprapubic pain with light spotting, which resolved spontaneously, and thereafter remained asymptomatic.

During a routine follow-up for a Papanicolaou (Pap) test at her local clinic six months later, the IUD strings were not visualized. A pelvic ultrasound at that time reported asymmetric uterine wall thickening, an embedded IUD extending from the endometrial cavity into the fundal myometrium without serosal breach, and features consistent with focal adenomyosis. Advanced imaging and intervention were not undertaken due to financial constraints related to limited medical insurance.

**Figure 1:**
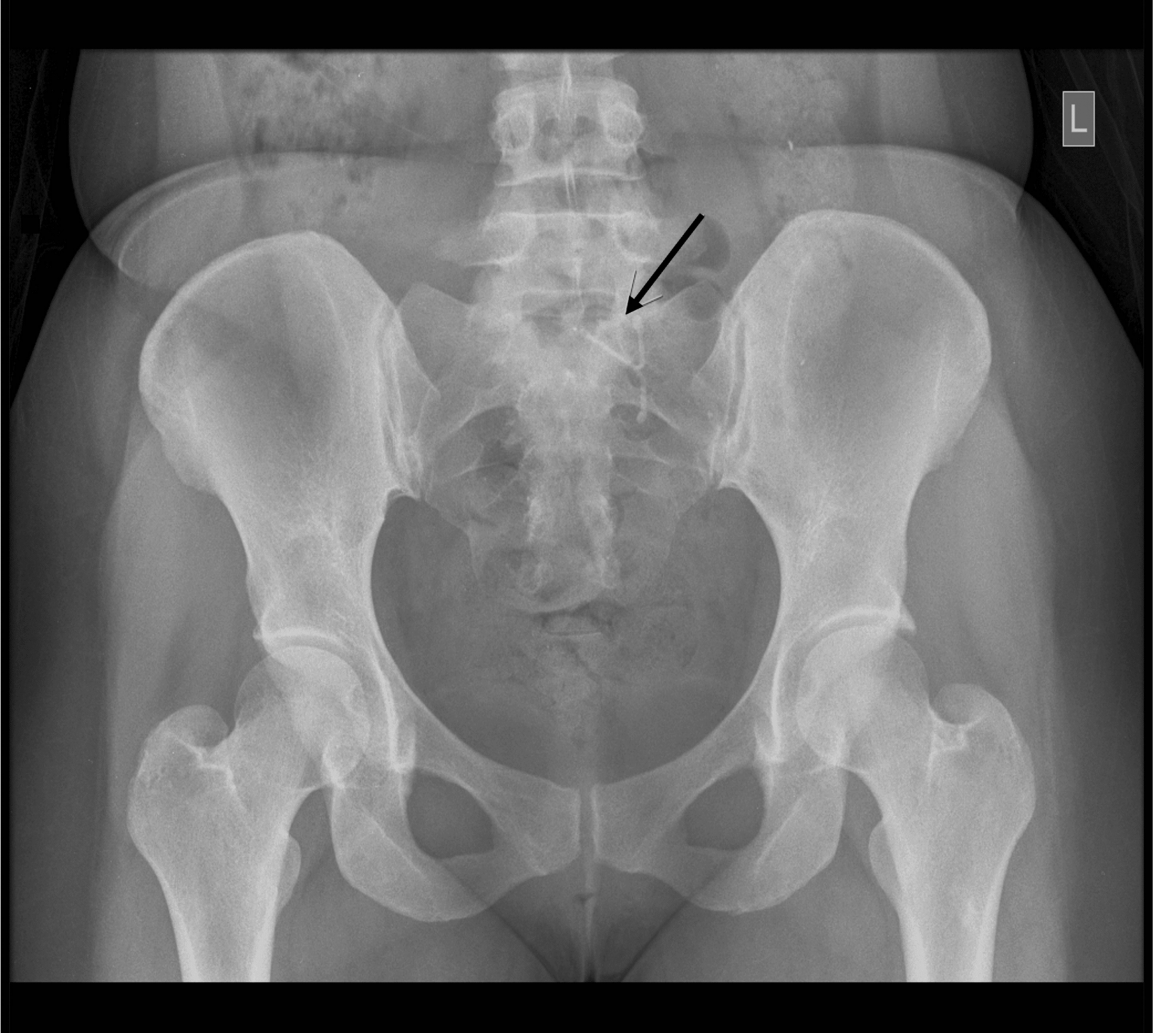
Plain pelvic radiograph showing the IUD (black arrow) located in the left pelvis in an extrauterine position. IUD: intrauterine device

**Figure 2:**
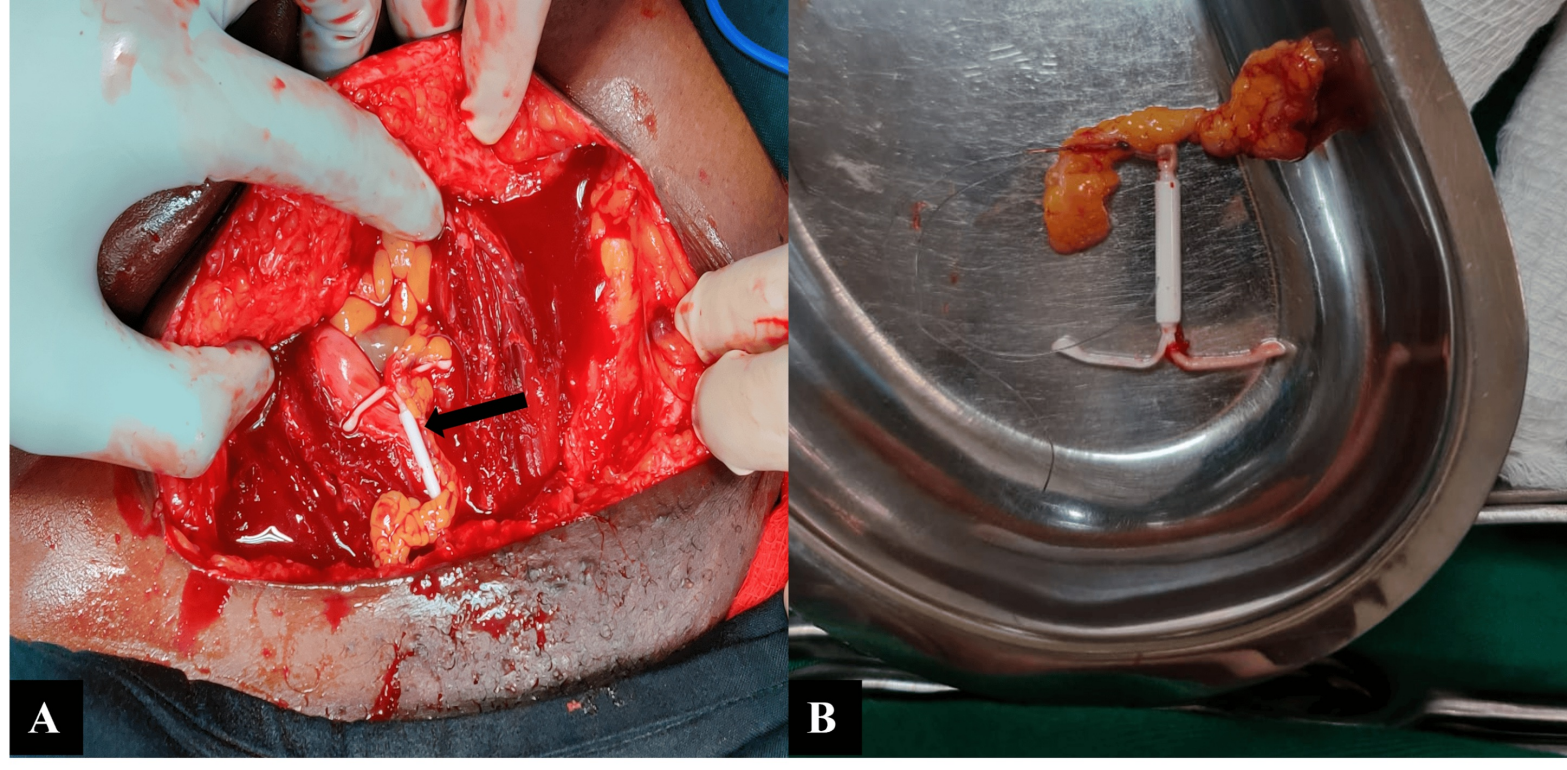
Intraoperative images showing the migrated IUD (black arrow) The IUD is seen adherent to the omentum on laparotomy (A) before successful retrieval (B). IUD: intrauterine device

Eleven months after insertion, she presented to our gynecology unit with the report from her previous scan, but no images. Repeat transabdominal ultrasound demonstrated focal adenomyosis, normal ovaries bilaterally, and an extrauterine IUD now located in the peritoneum, posterior to the left ovary. The pouch of Douglas and the urinary bladder appeared normal. A pelvic X-ray confirmed the presence of the IUD within the left pelvis in an extrauterine position (Figure 1). On examination, she only had localized tenderness in the left lower abdominal quadrant on deep palpation. After discussing possible management options and the associated risks, she opted for an exploratory laparotomy, as laparoscopy was not financially feasible. Informed consent was obtained, and the procedure was carried out electively. The laparotomy was undertaken via a Pfannenstiel approach following excision of the previous cesarean section scar. Intraoperatively, the uterus was bulky but otherwise normal, with intact adnexa and a clear pouch of Douglas. The IUD was identified behind the left ovary, partially adherent to omental tissue, without involvement of bowel, bladder, or other viscera (Figure 2A). It was carefully freed through partial omental cauterization and removed without complication (Figure 2B). The recovery was uneventful, and she was discharged after two days. She remained well and stable during long-term follow-up.

## Discussion

Uterine perforation following IUD insertion is a rare but potentially serious complication, occurring in approximately one to two per 1,000 insertions [4]. Primary perforation occurs within the first month of insertion, when the risk is highest, while secondary perforation develops later. Perforations may be partial, with the device embedded in the myometrium, or complete, in which the IUD penetrates the serosa and migrates into the peritoneal cavity or adjacent visceral organs [4,5]. Our patient’s initial ultrasound demonstrated partial perforation, which later progressed to complete perforation with extrauterine migration over five months.

Perforation and migration may be symptomatic, presenting with pelvic pain, abnormal bleeding, or missing strings; however, approximately 31% of cases are asymptomatic and identified only incidentally during imaging or examination [9]. As in our patient, asymptomatic migration poses a risk for delayed recognition and intervention, which increases the risk of visceral involvement, adhesions, and further translocation of the device.

Several risk factors for perforation have been reported, including high parity, insertion in the early postpartum period, lactation, and previous cesarean delivery [10]. Uterine pathology, such as adenomyosis or fibroids, may also alter myometrial architecture, making insertion more technically challenging and predisposing to abnormal embedment [10,11]. Our patient had both a history of cesarean section and sonographic features of adenomyosis, which likely contributed to the risk of perforation and migration. Studies show there is no clinically significant difference in the perforation risk between copper and levonorgestrel-releasing IUDs [10,12]. Ultimately, operator experience, uterine anatomy, and timing of insertion remain key determinants for uterine perforation [4].

The final location of migrated IUDs varies. While the intestine and bladder are frequent sites, with incidences of 32% and 24%, respectively, omental migration occurs in approximately 12% of cases [5,13-15]. Our case is unusual in that the IUD was found posterior to the left adnexa, adherent to the omentum, without injury to pelvic or abdominal viscera.

Management of migrated IUDs depends on location, patient symptoms, and available resources. Minimally invasive techniques such as laparoscopy or hysteroscopy are the gold standard, as they are associated with shorter hospital stays and fewer postoperative complications [7,16,17]. Laparotomy is typically reserved for cases with complex visceral involvement, dense adhesions, or where laparoscopy is unavailable [16]. In resource-limited settings such as ours, financial constraints and limited insurance coverage delayed diagnosis and ruled out laparoscopy, leaving laparotomy as the only practical option [18]. Despite these limitations, retrieval was successful, and recovery was uneventful.

## Conclusions

Uterine perforation is a serious complication of IUD insertion that may remain undetected, particularly in asymptomatic patients. This case underscores the importance of prompt evaluation when retrieval strings are not visualized and highlights how uterine pathology and prior cesarean section may predispose to device displacement. Awareness of the potential for silent migration is crucial, emphasizing the need for clinical vigilance and a low threshold for imaging in high-risk patients to facilitate early detection and optimal management.
